# Epigenetic silencing of tumor suppressor gene *CDKN1A* by oncogenic long non-coding RNA *SNHG1* in cholangiocarcinoma

**DOI:** 10.1038/s41419-018-0768-6

**Published:** 2018-07-03

**Authors:** Yang Yu, Mingjiong Zhang, Ni Wang, Quanpeng Li, Jian Yang, Shuai Yan, Xuezhi He, Guozhong Ji, Lin Miao

**Affiliations:** 1 Medical Center for Digestive Diseases, Second Affiliated Hospital, Nanjing Medical University, Nanjing, Jiangsu Province People’s Republic of China; 2 Department of Urology, Second Affiliated Hospital, Nanjing Medical University, Nanjing, Jiangsu Province People’s Republic of China; 3 Department of Oncology, The Affiliated Jiangyin Hospital of Southeast University Medical College, Jiangyin, Jiangsu Province People’s Republic of China; 4 Research Centre for Bone and Stem Cells, Nanjing Medical University, Nanjing, Jiangsu Province People’s Republic of China

## Abstract

Cholangiocarcinoma (CCA) is the as the most frequently observed biliary tract malignancy, which has low survival rate in addition to constrained treatment options; nevertheless, the fundamental molecular phenomenon underlying malignant progression of CCA is quite ambiguous. Recently long non-coding RNAs (lncRNAs) have been found to have significant regulatory functions in several human cancers. Herein, we have figured out that lncRNA *SNHG1*, with substantially enhanced expression in CCA, is capable of acting as the oncogenic molecule of CCA. As revealed by our data, *SNHG1* knockdown extensively inhibited CCA cell migration as well as proliferation in vitro and in vivo. In addition, in accordance with the findings of the RNA-Seq analysis, *SNHG1* knockdown exhibited a significant impact on the target genes that were linked to cell migration and regulation of cell proliferation, in addition to the apoptotic phenomenon. In a mechanistic manner, we also showed that *SNHG1* bound to the histone methyltransferase enhancer of the zeste homolog 2 (EZH2, which is regarded as the catalytic subunit of the polycomb repressive complex 2 (PRC2), which is an extremely conserved protein complex regulating gene expression with the help of methylating lysine 27 on histone H3), specifying the histone alteration pattern on the target genes, including* CDKN1A*, and, as a result, altered the CCA cell biology. These data verified a major function of the epigenetic regulation of *SNHG1* in CCA oncogenesis, in addition to its likely function as a target for CCA interruption.

## Introduction

Cholangiocarcinoma (CCA), the most frequently observed biliary tract cancer, encompasses 3% of all the gastrointestinal malignancies, in addition to being suggested as the malignancy that stems from ductal epithelial cells^1,2^. Improved knowledge of the carcinogenesis is critically important for the advancement of diagnostic markers, together with developing the innovative and productive therapies for CCA patients. The current modalities meant to establish a CCA diagnosis are insufficient, since it is still considered to be an uphill task to detect the ailment at an an early primary phase for enabling possibly therapeutic surgical treatments. Developing innovative biomarkers requires additional research on the DNA-methylation markers, in addition to peptide panels and non-coding RNAs.

Owing to the recent developments in deep-sequencing technologies, there are a number of previously unknown transcripts that have resulted in the identification. The majority (>99%) of these transcripts are regarded as long non-coding RNAs (lncRNAs), which are defined by their length of >200 nucleotides, which is found in a large number of RNA families; moreover, lncRNAs possess constrained protein-coding potential, while lacking the identifiable open reading frames, which are considered quite essential for assessing the protein-coding potential^3–7^. Recently, lncRNAs have been demonstrated to be capable of serving as pivotal regulators in numerous biological developments, for instance, cellular proliferation, development, differentiation, etc. Nonetheless, several lncRNAs have been uncharacterized; moreover, lncRNAs in CCA continue to a growing sphere of study, since few numbers of lncRNAs have been characterized in CCA tumorigenesis.

Abnormal expression of lncRNAs is likely to be involved in multiple aspects associated with human health and diseases, which include cancer, in particular, CCA^8–13,14^. Among them, small nucleolar RNA host gene 1 (*SNHG1*, also called *UHG*; *U22HG*;* lncRNA16*;* LINC00057*; *NCRNA00057*) has garnered our attention. Studying the invasive pathophysiology of *SNHG1* together with its overexpression is considered to be an effective predictor of oncogenesis in multifarious kinds of cancer, including esophageal squamous cell carcinoma^15^, lung squamous cell carcinoma^16^, hepatocellular carcinoma^17,18^, colorectal cancer^19,20^, and gastric cancer^21^. Nonetheless, finding out whether *SNHG1* is capable of serving as an “oncogene” in CCA is considered worthy enough.

In this research work, we have detected the lncRNA *SNHG1*, which, owing to its substantially enhanced expression in CCA, is capable of acting as an oncogenic molecule of CCA. In addition, *SNHG1* bound to the histone methyltransferase enhancer of the zeste homolog 2 (EZH2, which is a catalytic subunit of polycomb repressive complex 2 (PRC2), an extremely conserved protein complex regulating gene expression with the help of methylating lysine 27 on histone H3), followed by epigenetically suppressing *CDKN1A* (an inhibitor of the cyclin-dependent kinase, in addition to being a tumor-suppressive gene, which is used in treatment of several cancers^22–24^) expression in CCA cells, and, as a result, altered the CCA cell biology. Altogether, we shed light on the fact that *SNHG1* is capable of acting as an oncogenic molecule of CCA.

## Results

### *SNHG1* is upregulated in human CCA tissues

**Fig. 1:**
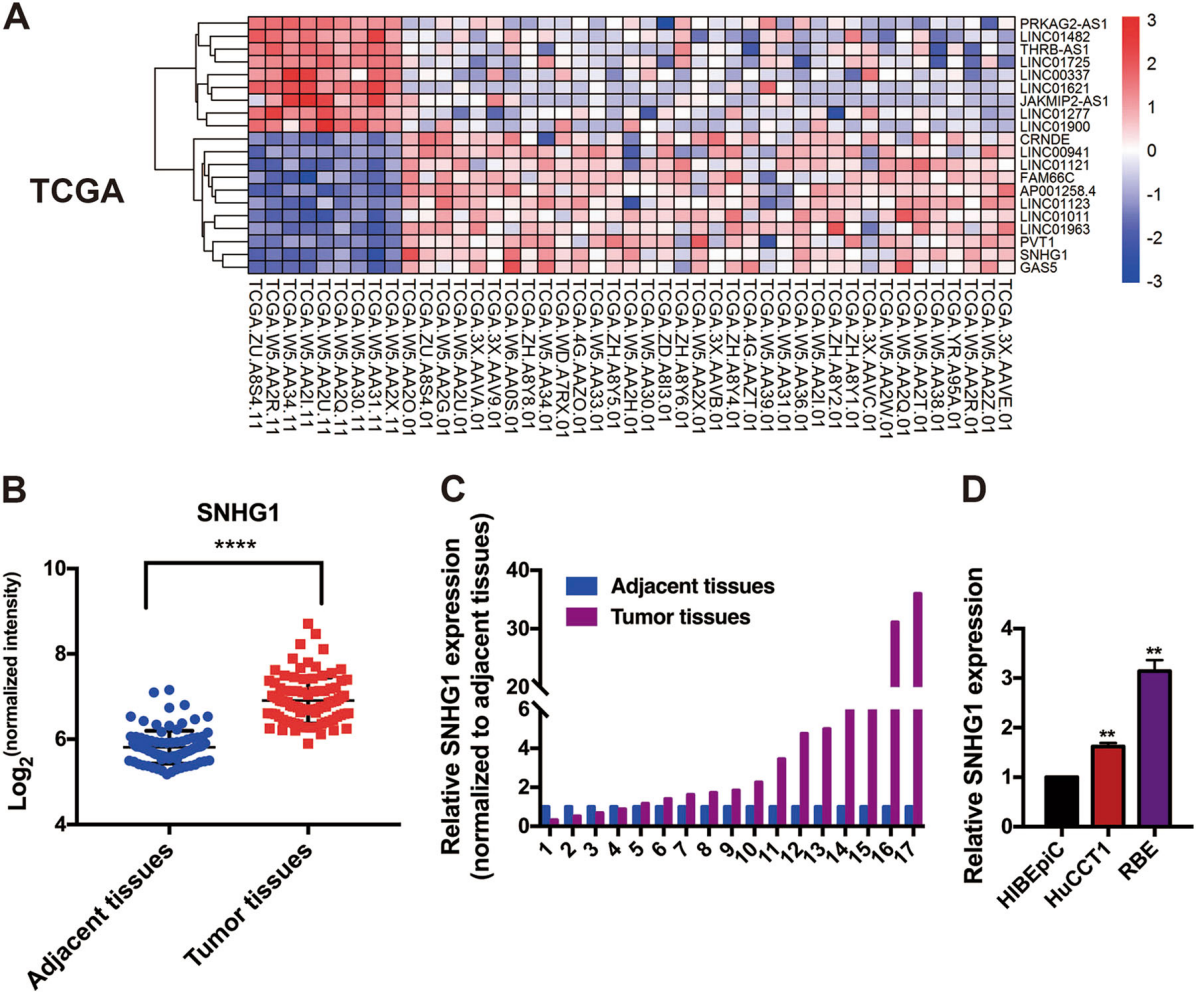
LncRNA *SNHG1* is overexpressed in cholangiocarcinoma (CCA) tissues. **a** Hierarchical clustering analysis of lncRNAs that were differentially expressed (fold change > 2; *P* < 0.05) in CCA tissues and normal tissues from TGCA database. **b** *SNHG1* is overexpressed in GEO datasets (GSE76297). **c** *SNHG1* was detected in 17 pairs of CCA tissues by qRT-PCR. The levels of *SNHG1* in CCA tissues are significantly higher than those in non-tumorous tissues. **d** *SNHG1* expression was analyzed by qRT-PCR in two CCA cell lines (HuCCT1 and RBE), compared with the normal human intrahepatic biliary epithelial cells (HIBEpiC). Error bars indicate means ± SD.**P < 0.01;****P < 0.0001.

When a detailed characterization of expressed lncRNAs in CCA, analysis of The Cancer Genome Atlas CCA, as well as typical tissue RNA-Sequencing data (including 9 normal as well as 36 cancer specimens) in addition to one independent microarray dataset from the Gene Expression Omnibus (GSE76297; 92 cancer tissue specimens as well as 91 normal tissue specimens), revealed the fact that *SNHG1* expression is augmented in tumor tissues in comparison with the nearby tissues (Fig. 1a, b). For the verification of the informatic data, *SNHG1* expression in a group consisting of 17 pairs of CCA tumor and adjacent tissues was figured out using quantitative real time -PCR (qRT-PCR), whereby it was validated that *SNHG1* exhibited remarkable expression levels in carcinoma tissues when compared with adjacent tissues (Fig. 1c). When compared with normal human intrahepatic biliary epithelial cells (HIBEpiC), *SNHG1* expression was higher in CCA cell lines (Fig. 1d). As suggested by these findings, *SNHG1* might have the potential to act as an “oncogene” for the promotion of CCA growth.

### Knockdown of *SNHG1* in CCA cell lines inhibits cell proliferation and migration

**Fig. 2:**
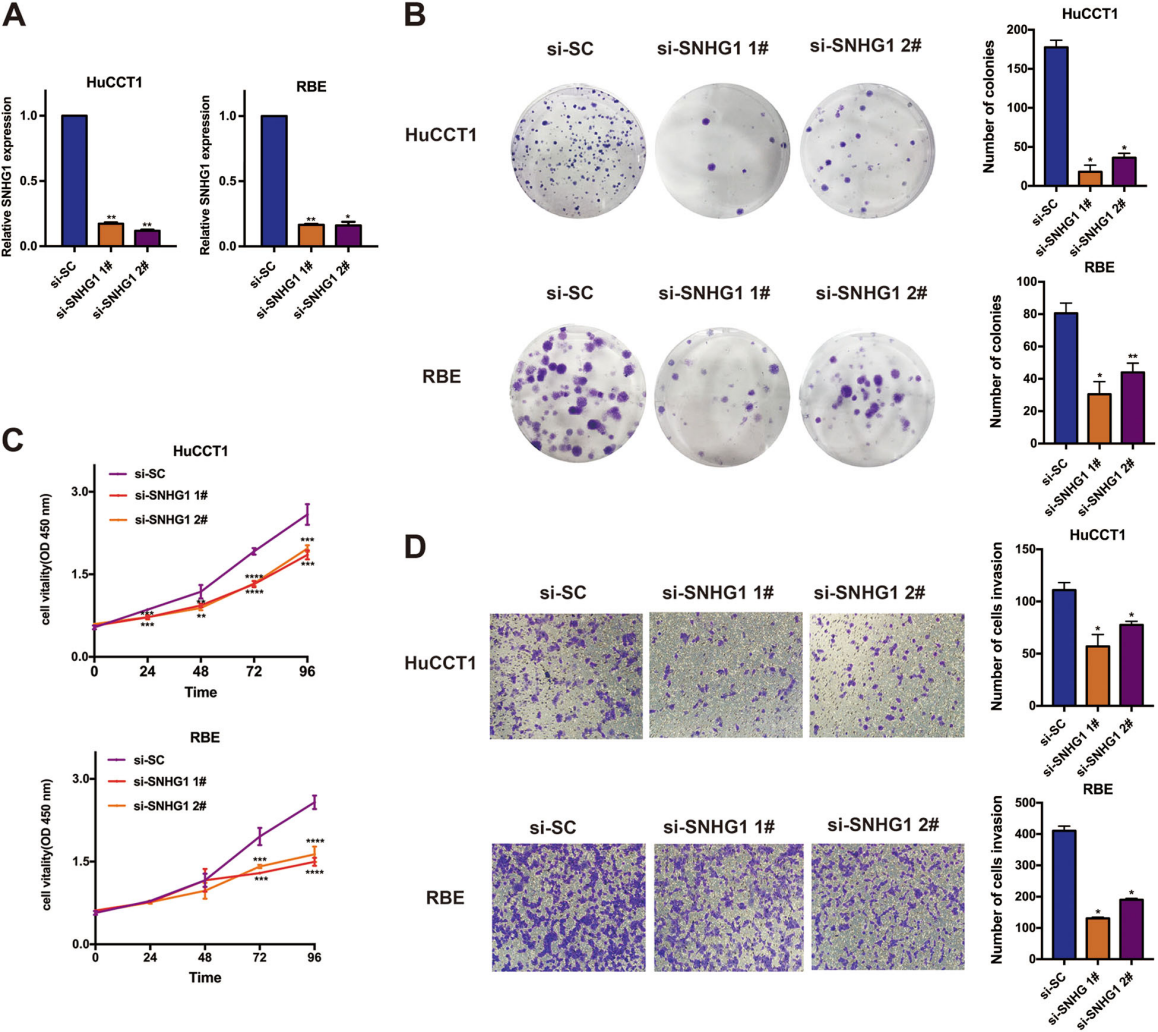
*SNHG1* promotes cell proliferation and migration in cholangiocarcinoma cells. **a** qRT-PCR detected the expression of* SNHG1* after siRNA transfection in HuCCT1 and RBE cell lines. **b** Colony formation assays were used to determine the cell colony formation ability of si-*SNHG1*-transfected cells. **c** CCK8 assays were used to determine the cell viability of si-*SNHG1*-transfected cholangiocarcinoma cells. **d** Transwell assays showed that *SNHG1* knockdown inhibited cholangiocarcinoma cell migration.Error bars indicate means ± SD. *P < 0.05; **P < 0.01;***P < 0.001;****P < 0.0001.

While investigating the impact of increased *SNHG1* in CCA, at first, as evident from Fig. 2a, qRT-PCR findings suggested that the expression of SNHG1 in the small interfering RNA (siRNA)-mediated knockdown group was substantially lowered, in comparison with the si-SC (scramble negative control) group, in the HuCCT1 and RBE cell lines (Fig. 2a). The clonogenic formation number exhibited a significant decline with the knockdown of *SNHG1* in the two cell lines (Fig. 2b). In addition to that, as suggested by the CCK8 assays, the knockdown of *SNHG1*expression disallowed the cell feasibility in HuCCT1 and RBE cell lines, when compared with the control cells (Fig. 2c). Thereafter, as revealed by the Transwell assays, the knockdown of *SNHG1* exhibited dramatic repression of the migration of cells (Fig. 2d). As suggested by these data, the *SNHG1* performs a quintessential function in CCA cancer cell proliferation as well as migration.

### Depletion of *SNHG1* leads to increased cell apoptosis and delayed cell cycle in CCA cell lines

**Fig. 3:**
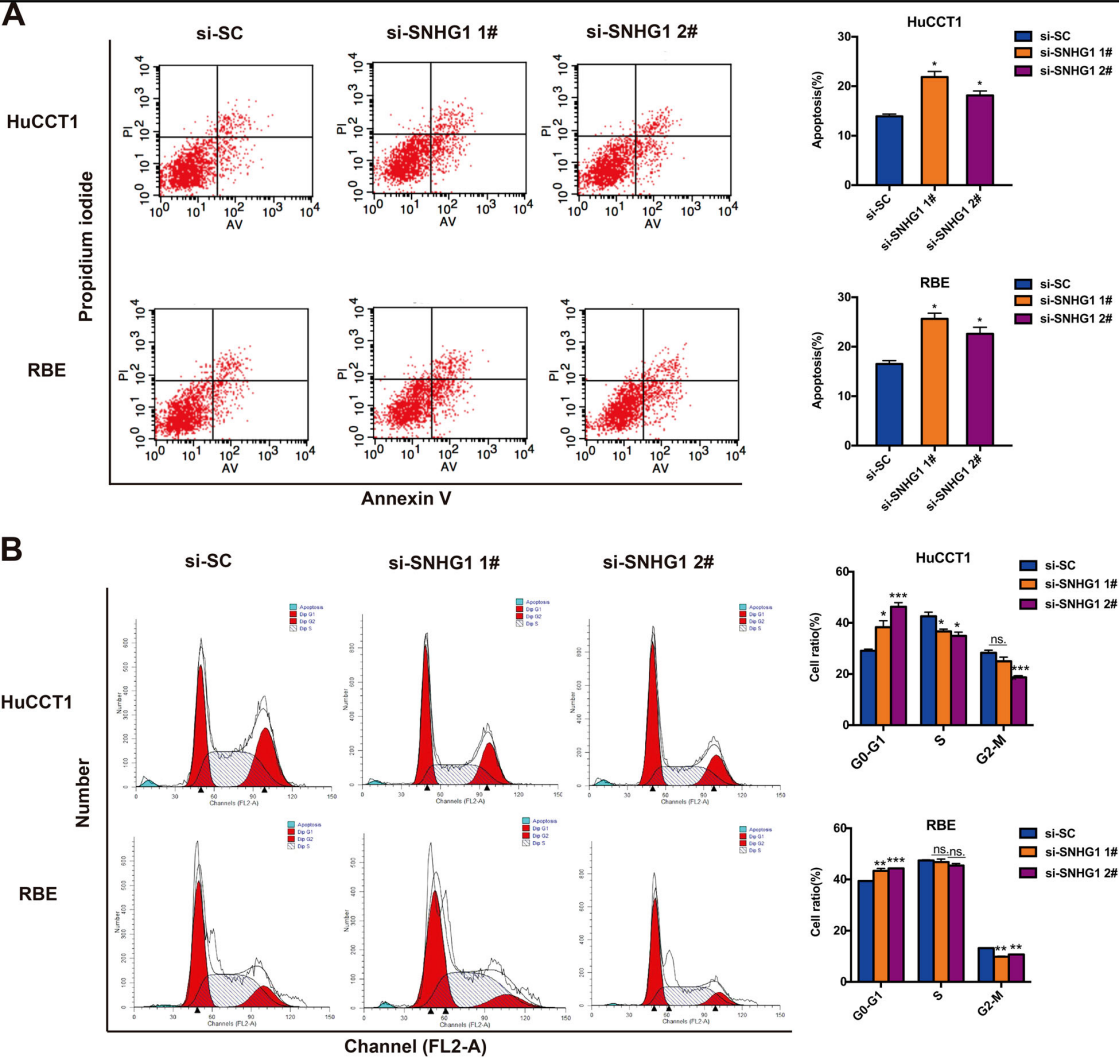
Knockdown of* SNHG1* causes apoptosis with the promotion of cell cycle arrest in vitro. **a** FACS analysis of the effect of *SNHG1* on cell apoptosis. **b** FACS analysis of the effect of *SNHG1*on cell cycle analysis. Error bars indicate means ± SD.*P < 0.05; **P < 0.01;***P < 0.001;n.s. not significant.

For the purpose of additionally investigating whether the *SNHG1* is capable of affecting the apoptosis of CCA cell lines, flow cytometry was carried out as well. As suggested by key observations, HuCCT1 and RBE cell lines transfected with *SNHG1* siRNA manifested higher apoptotic rate in comparison with the control group (Fig. 3a). Subsequent to that, for the purpose of deciding on whether the impacts of *SNHG1* on CCA cell proliferation and apoptosis are the key observations of the *SNHG1*-mediated alternations in the progression of cell cycle, we carried out the flow cytometry assay in HuCCT1 and RBE cell lines. As highlighted by the flow cytometry assay, the *SNHG1* knockdown gave rise to increased G0/G1 phase together with decreased S and G2/M phases, as compared with the control cells (Fig. 3b). Considered collectively, *SNHG1* is capable of accelerating cell proliferation, in addition to inhibiting apoptosis and regulating cell cycle progression of CCA cancer cell lines.

### Knockdown of *SNHG1* inhibits CCA cell tumorigenesis in vivo

**Fig. 4:**
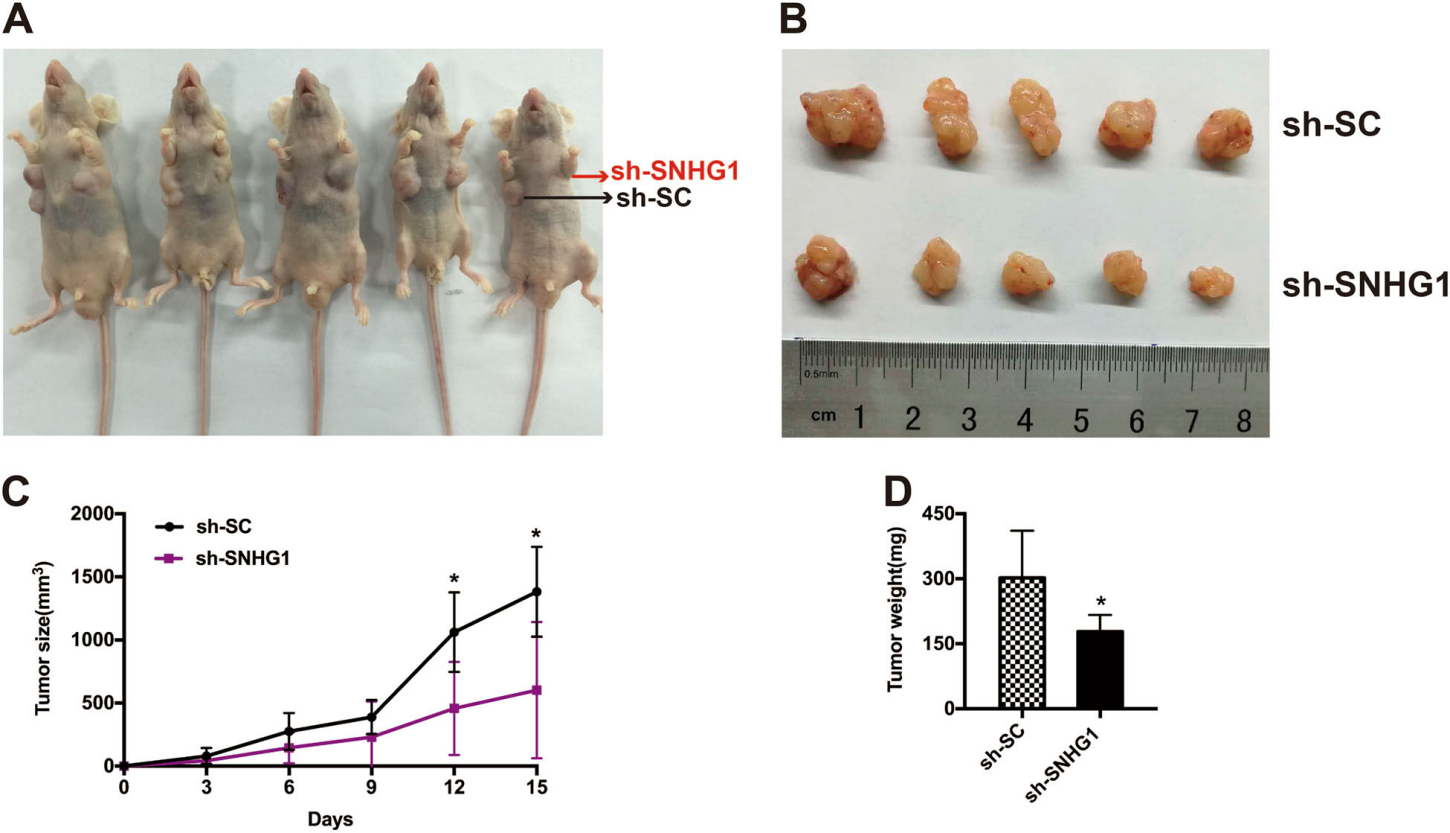
*SNHG1* regulates CCA cell proliferation in vivo. **a**, **b** Scramble or sh-*SNHG1*was stably transfected into HuCCT1 cells, which were injected into the nude mice. **c** Tumor volumes were calculated after injection every 4 days. Bars indicate standard deviation (S.D.). **d** Tumor weights are represented as means of tumor weights ± SD.*P < 0.05.

For the purpose of additionally confirming whether the *SNHG1* exerts an impact on the tumorigenesis of CCA in vivo, HuCCT1 cells transfected with sh-*SNHG1* or control vector were injected into nude mice. On the 16th day following the injection, tumors that developed in the sh-*SNHG1* cohort were remarkably smaller in comparison with those in the control cohort (Fig. 4a, b). Likewise, the average tumor volumes together with the respective weights in the ultimate experiment were apparently lower in the sh-*SNHG1*cohort as compared with the control vector cohort (Fig. 4c, d). As revealed by our findings, the silencing of *SNHG1* is capable of repressing the CCA tumor progression in vivo, suggesting the fact that *SNHG1* performs a substantial function in CCA tumor proliferation.

### Related target genes of *SNHG1* in CCA

**Fig. 5:**
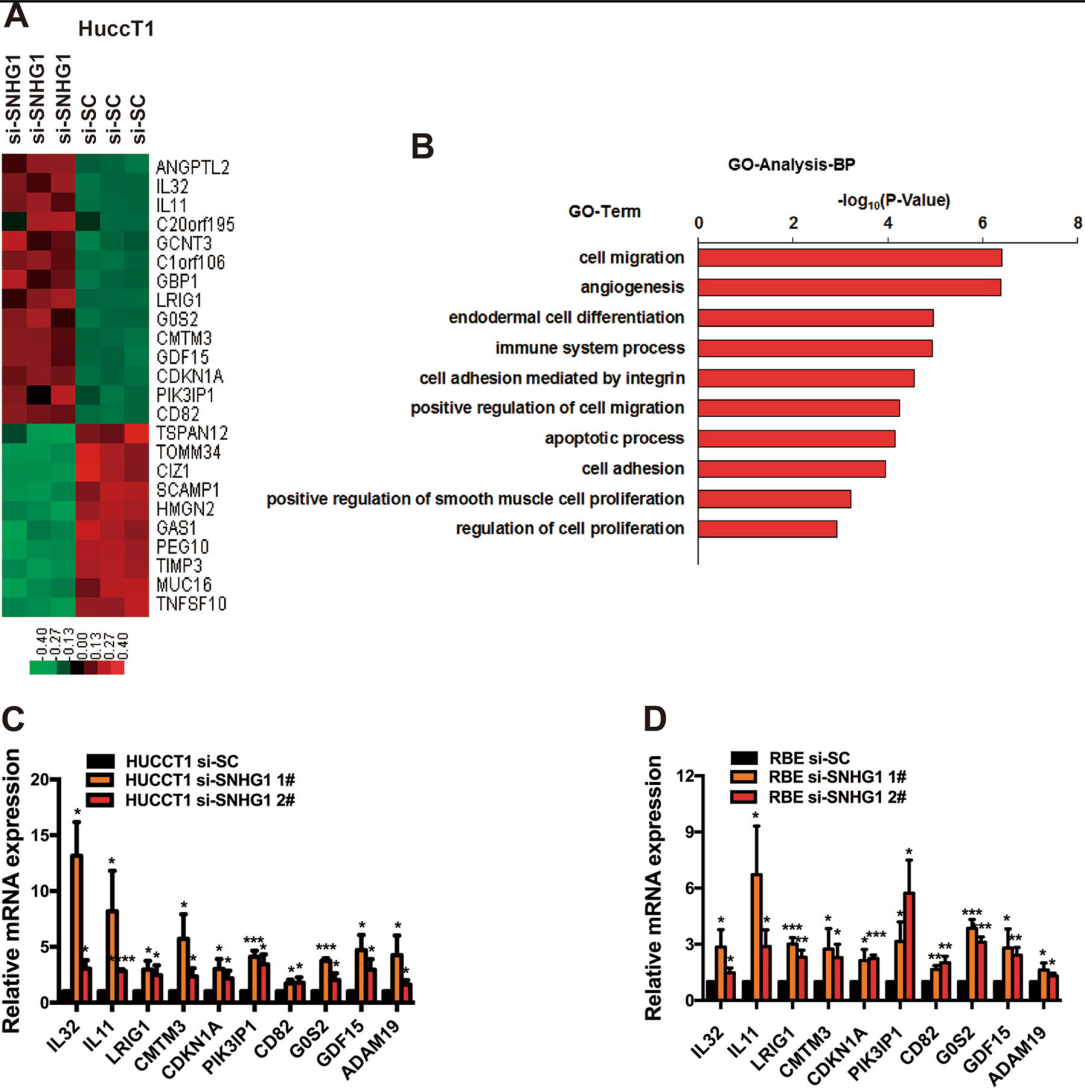
RNA-Seq after *SNHG1* knockdown in HuCCT1 cells. **a** Mean-centered, hierarchical clustering of 1295 transcripts altered (≥1.5-fold change) in siNC-treated cells and siRNA *SNHG1*-treated cells with three repeats. **b** Gene ontology analysis for all genes with altered expressions. **c**, **d** The altered mRNA levels of genes were selectively confirmed by qRT-PCR in knockdown* SNHG1*. Error bars indicate means ± SD.*P < 0.05; **P < 0.01;***P< 0.001;****P < 0.0001.

For the purpose of specifying the target genes that are capable of being regulated by *SNHG1* in CCA, RNA transcriptome sequencing was carried out in controls or siRNAs. A frequent set of 971 mRNAs revealed ≥1.5-fold increased abundance; conversely, 546 genes exhibited a decline in abundance (≤1.5-fold) owing to the silencing *SNHG1* (Fig. 5a, Supplementary Table S2). A thorough research of the ontology analysis highlighted the most distinct over-represented biological phenomena with the involvement of pathways in cell migration together with regulation of cell growth and apoptotic phenomenon (Fig. 5b). For the purpose of prioritizing most *SNHG1*-associated genes, attention was given to the genes that were most extensively expressed with knockdown of *SNHG1*. Prospectively, among the most highly expressed genes, a number of renowned genes associated with proliferation and migration (e.g., *IL3*2,* IL11*, *LRIG1*, *CMTM3*, *CDKN1A*, *PIK3IP1*, *CD82*, *G0S2*, *GDF15*, and *ADAM19*, et al.) are included. Verification of some of these genes was carried out using qRT-PCR, subsequent to the knockdown of *SNHG1* in HuCCT1 and RBE cells (Fig. 5c, d).

### *SNHG1* epigenetically silenced *CDKN1A* transcription through EZH2-mediated H3K27me3 demethylation

**Fig. 6:**
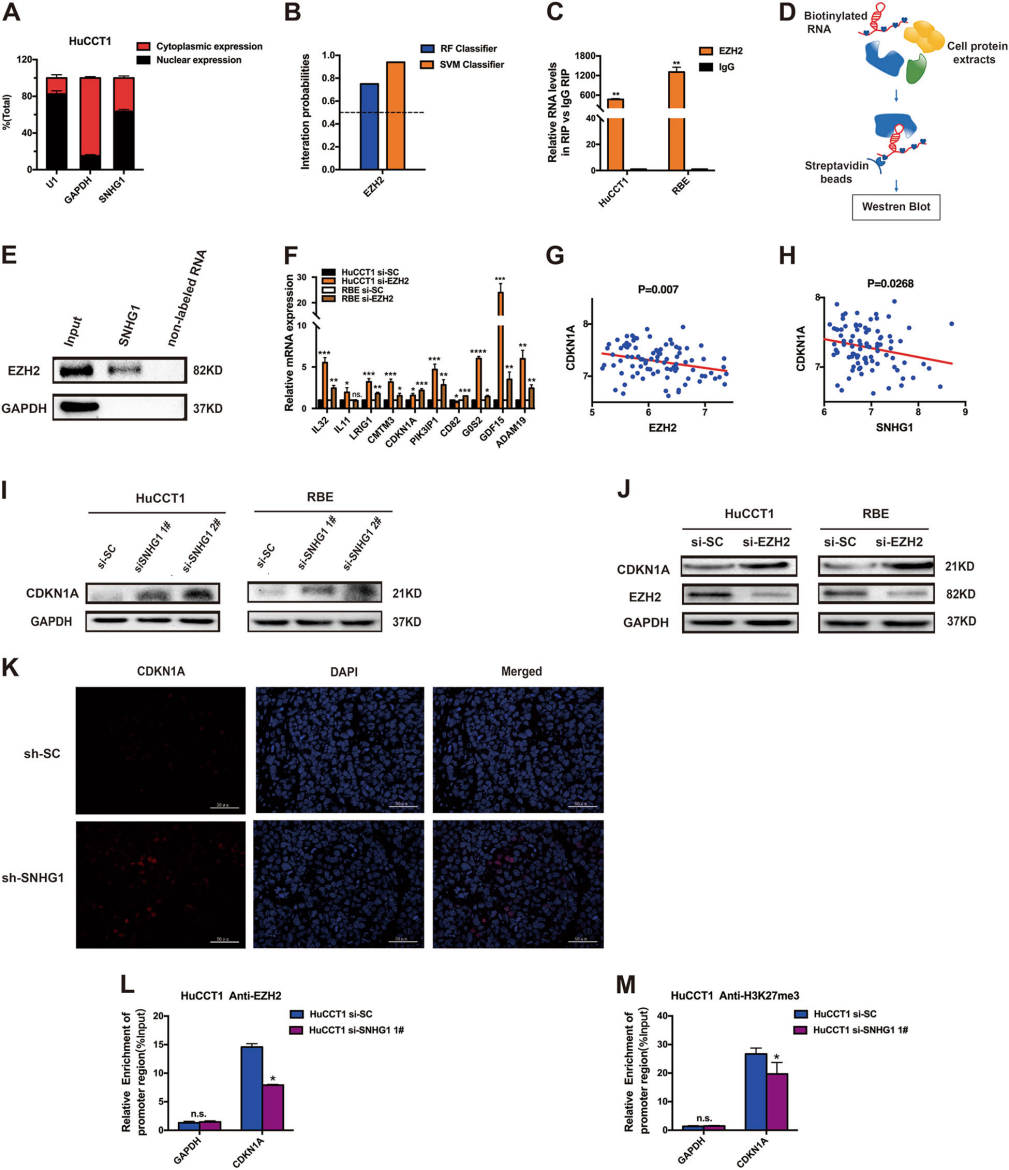
*SNHG1* binds with EZH2 in the nucleus, thus leading to epigenetical silencing of *CDKN1A*. **a** After nuclear and cytosolic separation, RNA expression levels were measured by qRT-PCR. GAPDH was used as a cytosol marker and U1 was used as a nucleus marker. **b** The interation probabilies of EZH2 and *SNHG1* were detected on the website, and the results showed that EZH2 could well bind with *SNHG1* well (http://pridb.gdcb.iastate.edu/RPISeq/index.html). **c** RIP experiment for EZH2 was performed and the co-precipitated RNA was subjected to qRT-PCR for *SNHG1*. **d** Schematic of the RNA pull-down experiment for the identification of proteins associated with* SNHG1*. **e** In vitro transcribed, pull-down assays showed that desthiobiotinylated* SNHG1* could retrieve EZH2 in HuCCT1 cells, but not GAPDH. GAPDH was a negative control. **f** The methylation-related genes were detected by qRT-PCR in HuCCT1 and RBE cell lines after knockdown of *EZH2*. **g** The correlation between *EZH2* and *CDKN1A* expression was detected by analyzing GSE76297 data. **h** The correlation between *SNHG1* and *CDKN1A* expression was detected by analyzing GSE76297 data. **i** The altered protein levels of CDKN1A were selectively confirmed by western blot in knockdown of *SNHG1*. **j** The altered protein level of CDKN1A was selectively confirmed by western blot in knockdown *EZH2*. **k** Immunofluorescence was done to explore if the expression of CDKN1A was changed by knockdown of* SNHG1* in vivo. **l**, **m** ChIP of EZH2 and H3K27me3 of the promoter region of *CDKN1A* locus after siRNA treatment targeting si-SC and si-*SNHG1* 1# in HuCCT1 cells; qPCR was performed to detect the quantitation of ChIP assays. Enrichment was quantified relative to input controls. Antibody directed against IgG was used as a negative control. Error bars indicate means ± SD. *P < 0.05; **P < 0.01;***P < 0.001;****P < 0.0001; n.s. not significant.

There are reports that a number of lncRNAs have been validated for working through the co-operation with chromatin-modifying enzymes for the purpose of accelerating epigenetic activation and, accordingly, silencing of the target gene expression^25^. In particular, PRC2, which is a classical methyltransferase, constituted with EZH2, in addition to EED and SUZ12, is capable of serving as a catalyst not only in the di- but also in the trimethylation of lysine residue 27 of histone 3 (H3K27me3), and, accordingly, performing epigenetic repression of the expression of the target genes^26,27^. In our research work, for the purpose of investigating the system for* SNHG1*-mediated regulation, first, subcellular fractionation location assays were performed to verify the localization of *SNHG1* mainly in the nucleus mainly (Fig. 6a). Additionally, the interation probabilies of EZH2 and *SNHG1* were identified on the website (http://pridb.gdcb.iastate.edu/RPISeq/index.html); moreover, as revealed by the findings, EZH2 is capable of binding with the *SNHG1* well (Fig. 6b). As evident from Fig. 6c, amplification of endogenous *SNHG1*was observed in the anti-EZH2 RNA immunoprecipitation (RIP) fraction associated with the input, when compared with the IgG fraction in the HuCCT1 and RBE cell lines. Then, RNA pull-down assays demonstrated that labeled *SNHG1* RNA, but not non-labeled RNA, specially retrieved EZH2 from HuCCT1 nuclear extract (Fig. 6d, e). Considered collectively, these results validated the fact that *SNHG1* was capable of interacting with EZH2.

Subsequent to that, we tested whether EZH2 was capable of coregulating the suppression of these *SNHG1*-suppressed genes through a bond with *SNHG1*. Primarily, we investigated the expression of* SNHG1*-suppressed genes with by knockdown or not of *EZH2*, using RT-qPCR. In addition, as revealed by the findings, the *SNHG1*-suppressed genes exhibited an increase through the knockdown of *EZH2*, as well (Fig. 6f), in the HuCCT1 and RBE cell lines.

Correlation analysis of the dataset GSE76297 (92 pairs of cancer and 91 normal tissue samples) from the Molecular Signature Database suggested that *CDKN1A* was substantially negatively correlated with *EZH2* (Fig. 6g) and *SNHG1* (Fig. 6h). In addition, the protein level of CDKN1A exhibited an increase through the knockdown of *SNHG1* (Fig. 6i) and *EZH2* (Fig. 6j). Simultaneously, immunofluorescence was done to explore whether the expressions of EZH2 and CDKN1A were changed with the knockdown of *SNHG1* in vivo. The results showed that when *SNHG1* was knocked down the expression of CDKN1A was improved (Fig. 6k), while the expression of EZH2 showed no obvious change (Figure S1 A).

To summarize, we shed light on one of the coregulating genes by *SNHG1* as well as EZH2 in CCA, *CDKN1A*, based on the report that the hypermethylation of the *CDKN1A* promoter region contributed to *CDKN1A* transcription inactivation in breast cancer^28^. In addition, as revealed by more and more evidences, EZH2 directly targets the *CDKN1A* promoter in the neural progenitors; moreover, its activity exhibits correlation with the modifications in the H3K27me3 levels^29^. The *EZH2* blockade by RNA interruption hampers the proliferation of the ovarian cancer through the facilitation of re-expression of *p21*^30,31^.

Furthermore, to additionally determine whether *SNHG1* suppressed the expression of *CDKN1A* through interaction with the EZH2, chromatin immunoprecipitation (ChIP) analysis was conducted. As suggested by the ChIP assays, knockdown of *SNHG1* lowered the binding of EZH2, together with the H3K27me3 levels all through the promoters of *CDKN1A* (Fig. 6l, m). These findings validated the fact that EZH2 is capable of directly binding to the promoter of *CDKN1A*, followed by repressing the *CDKN1A* expression directly through mediation of the H3K27me3 demethylation modification.

As discovered by our findings, knockdown of the *SNHG1* lowered the binding of EZH2 as well as H3K27 trimethylation levels all through the promoter of the *CDKN1A*, resulting in the augmented level of the *CDKN1A* that was capable of decelerating the growth of CCA. As suggested by our findings, *SNHG1* promotes CCA malignancy through a bond with EZH2, followed by repressing the expression of the *CDKN1A* epigenetically in the nucleus.

### *CDKN1A* is a bona target of *SNHG1*, and *CDKN1A* overexpression suppresses CCA cell proliferation and metastasis

*CDKN1A*, considered to be among the most CKIs, is an integral checkpoint of the *P53* signaling pathway with respect to G1/S transition through the inhibition of the function of kinases, for instance, CyclinD/CDK4, CyclinD/CDK6, as well as CyclinE/CDK2^32,33^, playing different kinds of function to inhibit the cell progression in typical as well as cancer cells, in addition to being downregulated in various cancers^34–37^. However, no report has proved that *CDKN1A* is a tumor suppression gene of CCA.

**Fig. 7:**
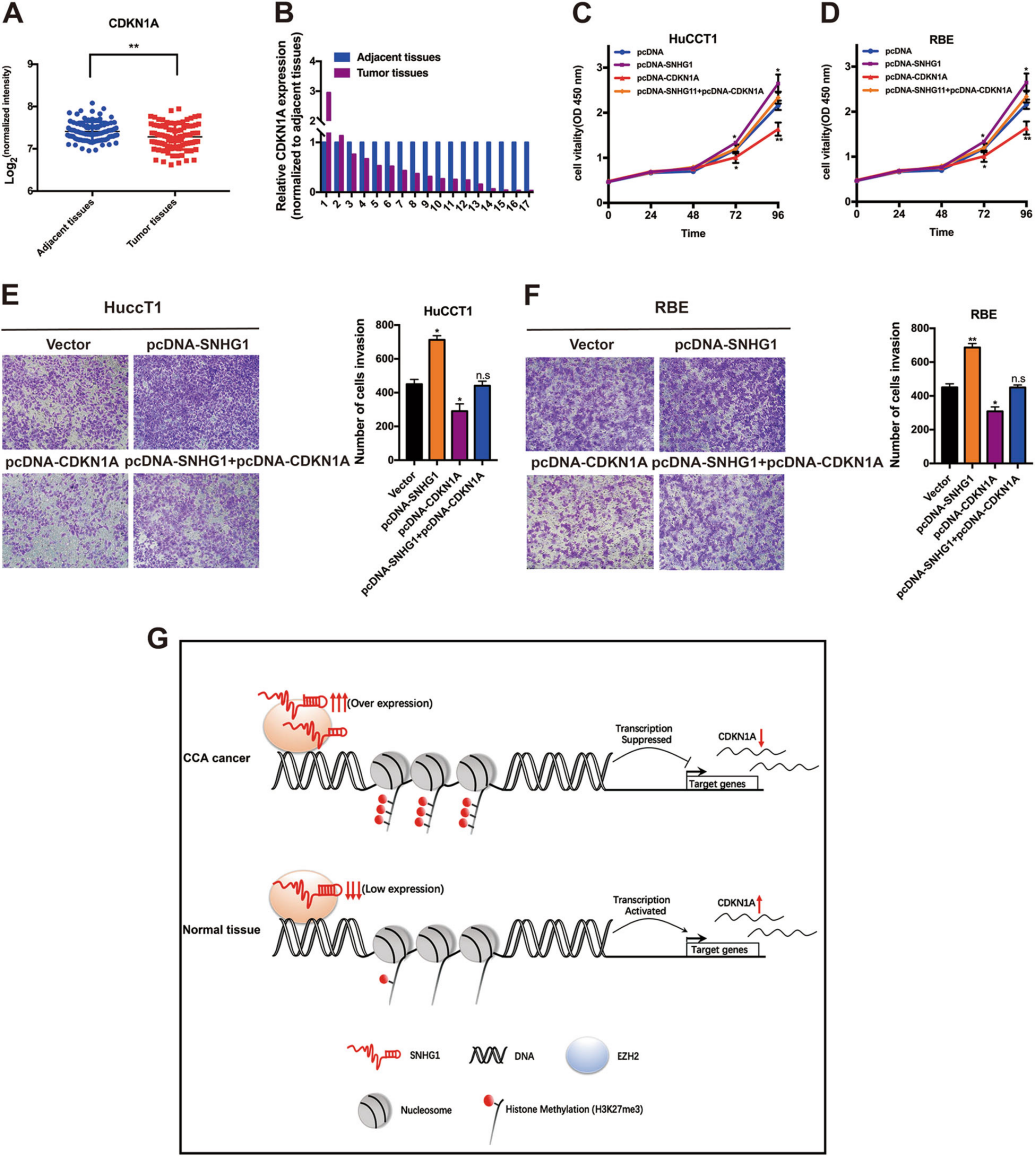
*CDKN1A* is a bona target of *SNHG1*, and *CDKN1A* overexpression suppresses CCA cell proliferation and metastasis. **a** Expression level of *CDKN1A* in cholangiocarcinoma by analysis of GES76297 data. **b** *CDKN1A* was detected in 17 pairs of CCA tissues by qRT-PCR. **c**–**f** HuCCT1 and RBE cells transfected with vector/pcDNA-*CDKN1A*/pcDNA-*SNHG*1 and with *SNHG1* followed by transfection with *CDKN1A*. After transfection, cells were analyzed by CCK8 assays (**c**, **d**) and Transwell assays (**e**, **f**). **g** Proposed model of *SNHG1* regulating *CDKN1A* expression to promote CCA tumor growth. Error bars indicate means ± SD. *P < 0.05; **P < 0.01; n.s. not significant.

First, for the assessment of *CDKN1A* expression in CCA tissues, we carried out the analysis of the dataset GSE76297 (92 pairs of cancer and 91 normal tissue samples) and figured out that *CDKN1A* had lower expression in the cancer tissues in comparison with the typical tissues in CCA (Fig. 7a). Subsequent to that, by qRT-PCR, we found that the *CDKN1A* expression is lower in CCA tumor tissues in comparison with the neighboring ones through the detection of the expression in a cohort of 17 pairs of CCA tumor tissues in comparison with the nearby tissues using qRT-PCR (Fig. 7b). In addition, we also found that overexpression of *CDKN1A* is capable of substantially suppressing proliferation and invasion of HuCCT1 and RBE cell lines; moreover, overexpression of *CDKN1A* is capable of partially reversing the *SNHG1*-mediated progression and migration promotion (Fig. 7c–f).

In summary, our study demonstrated the regulatory mechanism in tumorigenesis of *SNHG1*, by which *SNHG1* could promote malignancy of CCA by binding to histone methyltransferase EZH2 (the catalytic subunit of the PRC2, a highly conserved protein complex that regulates gene expression by methylating lysine 27 on histone H3), and then specified the histone modification pattern on the target genes, including *CDKN1A*, thus explaining cell survival and metastasis of CCA (Fig. 7g).

## Discussion

During the previous decades, more and more evidence has laid emphasis on the rising importance of lncRNAs in diversified human cancers, which include CCA^9–12,14^. LncRNAs exhibit an evident benefit associated with their relative tissue-specific expression, as well as the functional structure at the transcriptional levels. Nevertheless, lncRNAs in CCA are still considered to be part of a growing sphere, since there are just few lncRNAs that have been linked to CCA tumorigenesis, requiring further investigations to be considered. By this study we have discovered that the expression of *SNHG1*, an lncRNA, leads to substantial upregulation of the CCA tissues.

The dysregulation of lncRNAs is linked with an extensive array of pathological mechanisms; conversely, the mechanisms of lncRNA expression are quite unclear and require additional investigation. As indicated by our data, knockdown of *SNHG1* expression substantially inhibits cell growth in vitro and vivo. Moreover,* SNHG1* has manifested oncogenic attribute in different kinds of cancers; nevertheless, the genes influenced by the *SNHG1* continue to remain uncertain. With the help of RNA-Seq, we not only observed the fact that the findings of gene ontology analysis were, in particular, associated with cell migration and regulation of cell progression, together with apoptotic mechanism, but also figured out several target genes that were regulated by the* SNHG1*. The latest research works have highlighted that some lncRNAs perform the epigenetic regulation of gene expression with the help of DNA methylation as well as histone modifications, containing the methylation, in addition to functions such as acetylation and phosphorylation^38–40^. As discovered by our findings, *SNHG1* is capable of interacting with EZH2, which is considered to be a type of histone methylation modification complex in the nucleus, thus regulating an array of the target gene’s expression, as shown by the mounting evidences.

*CDKN1A*, one of the genes regulated by *SNHG1* in accordance with the RNA-Seq, is capable of exhibiting the role of tumor suppressor genes in different kinds of cancer^35–37^. Furthermore, there are reports that hypermethylation of the *CDKN1A* promoter region contributes to the *CDKN1A* transcription inactivation in breast cancer^28^. Nevertheless, the function of *CDKN1A*in the tumorigenesis of CCA continues to be uncertain. As discovered by our findings, *SNHG1*is capable of binding to EZH2, which is a kind of histone methylation modification complex in the nucleus, thus suppressing the target gene’s expression, which includes* CDKN1A*, an innovative tumor suppressor in CCA.

To summarize, as suggested by our research works, the regulatory mechanism in the tumorigenesis of *SNHG1*, by which* SNHG1* could promote malignancy of CCA through regulating the transcription of *CDKN1A* epigenetically in the nucleus, facilitates cell survival and metastasis of CCA. Ultimately, fast-paced developments in the oligonucleotide/nanoparticle technology bring forth optimism for the delivery of siRNA-oriented therapeutics for the regulation of the lncRNA levels in vivo. As highlighted by our key observations, there is a need of additional research dealing with the capability of* SNHG1* as an informative biomarker as well as a therapeutic target in patients with CCA.

## Materials and methods

### Tissue gathering and ethics statement

Altogether 17 samples were analyzed in this research, and all were subjected to resection of the CCA at the Second Affiliated Hospital of Nanjing Medical University. All the specimens were instantly frozen in tubes with RNAlater preservation liquid after being removed and were kept in liquid nitrogen till the extraction of RNA. Our research was permitted by the Ethics Committee of Nanjing Medical University (Nanjing, Jiangsu, PR China) with written consent from every patient.

### RNA extraction as well as qRT-PCR analyses

All RNAs were obtained from the cultured cells or specimens with TRIzol reagent (Invitrogen, Carlsbad, CA, USA). For RT-qPCR, 1 μg of RNA was reverse transcribed with cDNA using a Reverse Transcription Kit (Takara, Tokyo, Japan). Real-time PCR analyses were carried out with SYBR Green (Takara, Tokyo, Japan). The findings were regulated to the expression of glyceraldehyde-3-phosphate dehydrogenase (GAPDH). The primer sequences are shown in Supplementary Table S1.

### Cell culture

CCA cell lines (HuCCT1 and RBE) and normal HIBEpiC were obtained from the Institute of Biochemistry and Cell Biology of the Chinese Academy of Sciences (Shanghai, China). All the cell lines were maintained in Dulbecco’s modified Eagle medium (Life Technologies Corporation Attn, Grand Island, USA) coupled with 10% fetal bovine serum (FBS; Sciencell, Carlsbad, CA), 100 mg/ml streptomycin, and 100 U/ml penicillin (Invitrogen, Shanghai, China) in humidified air at 37 °C with 5% CO_2_.

### Transfection of cell lines

CCA cells were planted at six-well plates as well as transfected with particular siRNA (100 nM) or scramble negative control siRNA (100 nM) the next day using Lipofectamine 2000 (Invitrogen, Carlsbad, CA, USA) on the basis of the producer’s protocol. Scrambled negative control siRNA (si-SC) was bought from Invitrogen (Invitrogen, Carlsbad, CA, USA). sh-*SNHG1* was cloned into pENTRTM/U6 vector; and the orders of siRNAs and shRNA are summed up in Supplementary Table S1.

### Subcellular fractionation address

The division of nuclear as well as cytosolic fractions was constructed with the PARIS Kit (Life Technologies, Carlsbad, CA, USA) following the producer’s guides.

### Cell proliferation analysis

Cell viability was monitored with CCK8 kit (Houston TX, USA) following the producer’s suggestions. The HuCCT1 and RBE cells transfected with si-*SNHG1* or si-SC (scramble negative control) (3000 cells/well) were cultivated in five 96-well plates with six replicate wells. In terms of the colony formation assay, altogether 500 transfected cells were addressed in a 6-well plate and kept in a medium with 10% FBS for 2 weeks with the replacement of the medium every 4 days. Then, colonies were confirmed with methanol and dyed with 0.1% crystal violet (Sigma-Aldrich, St Louis, MO) for 15 min. The quantity of visibly stained colonies was counted in colony formation. In different treatment groups, wells were independently measured in triplicate.

### Assays of cell migration

In terms of the migration assays, after 24 h of transfection, 3.5 × 10^4^ cells in medium with 1% FBS were put into the upper chamber of an insert (Millipore, Billerica, MA, USA), while medium with 10% FBS was put into the lower chamber. After 24 h of incubation, the remaining cells on the upper level of the membrane were cleaned, while those cells that had migrated through the membrane were dyed with methanol as well as 0.1% crystal violet and then imaged with an IX71 inverted microscope (Olympus, Tokyo, Japan). The experiments were done in triplicate.

### Flow cytometry analysis

Flow cytometry assays were performed as previously reported by Xu et al.^41^. After the cells were transfected with siRNAs for 48 h, we harvested the cells and then performed fluorescein isothiocyanate (FITC)-Annexin V and propidium iodide staining by using the FITC-Annexin V Apoptosis Detection Kit (BD Biosciences, Franklin Lakes, NJ, USA) according to the manufacturer’s instruction. Cell cycle analysis was stained with propidium oxide by the Cycle TEST PLUS DNA Reagent Kit (BD Biosciences) by following the manual and then evaluated by FACScan. The count of the cells in each phase was assessed.

### Western blot assay as well as antibodies

Cell protein lysates were divided by 10% sodium dodecyl sulfate-polyacrylamide gel electrophoresis, transferred to 0.22-m nitrocellulose membranes (Sigma-Aldrich, St Louis, MO), and cultivated with particular antibodies. Densitometry (Quantity One software; Bio-Rad) was used to quantify the autoradiograms. GAPDH antibody was employed as control. Anti-EZH2 was from proteintech (Wuhan, China) and anti-CDKN1A was from Abcam (Cambridge, UK).

### In vivo tumor formation assay

Athymic male mice, 4 weeks old and bought from the Animal Center of the Nanjing University (Nanjing, China), were kept in particular in pathogen-free situations. HuCCT1 cells were stably transfected with shRNA or empty vector and obtained from cell culture plates, cleaned with phosphate-buffered saline, and re-suspended at 2 × 10^7^ cells/ml. The cells were then xenografted into BALB/c male nude mice. Besides, the size of the tumor, length × width^2^ × 0.5, was calculated every 3 days. At 16 days post injection, the mice were sacrificed by CO_2_ asphyxiation and the tumor weights were weighed and examined. The study was performed in coincidence with the Instruction for the Care and Use of Laboratory Animals of the National Institutes of Health strictly. The agreement was licensed by the Committee on the Ethics of Animal Experiments of Nanjing Medical University.

### Deep sequencing of whole transcriptome

Total RNA from the HuCCT1 cells with *SNHG1* knockdown as well as control HuCCT1 cells were separated and quantified. The concentration of each specimen was measured with NanoDrop 2000 (Thermo Scientific, USA). The amount was evaluated with Agilent2200 (Agilent, USA). The ordering library of each RNA specimen was carried out with Ion Proton Total RNA-Seq Kit v2 following the conditions recommended by the manufacturer (Life technologies, USA). Data can be accessed in Supplementary Table S2.

### RIP assays

RIP experiment was carried out to study whether *SNHG1* could interact with EZH2, using a Magna RIP^TM^ RNA-Binding Protein Immunoprecipitation Kit (Millipore, Billerica, MA, USA) following the manufacturer’s guidelines. The antibody used for RIP assays of EZH2 was obtained from Millipore (Billerica, MA, USA).

### ChIP assays

ChIP assays were carried out with EZ-CHIP Kit following the manufacturer’s recommendations (Millipore, Billerica, MA, USA). EZH2 antibody was purchased from Millipore (Billerica, MA, USA) and H3 trimethyl Lys 27(H3K27me3) antibody was from Abcam (Cambridge, UK). The sequences of ChIP primer are shown in Supplementary Table S1. We calculated the ChIP data as a proportion of the added DNA using the equation _2_[input Ct − target Ct] _× 0.1 × 100_.

### Immunofluorescence

CCA cells were seeded on chamber slides and were fixed with 4% paraformaldehyde for 10 min at room temperature. Then, the cells were incubated with antibodies against EZH2 (proteintech, Wuhan, China) or CDKN1A (Abcam, Cambridge, UK) at 4 °C overnight. Then, the slides were incubated with matched secondary antibodies at room temperature for 50 min in dark condition. The nuclei of the cells were stained with 4′,6-diamidino-2-phenylindole (Sigma) at room temperature for 10 min. Fluorescence images were captured using a Pannoramic MIDI/250 (3D HISTECH, Hungary).

### In vitro transcription assays and RNA pull-down assays

In vitro translation assays were performed using mMessage mMachine KIT following the manufacturer’s recommendations (Ambion, USA).* SNHG1* RNAs were labeled by desthiobiotinylation, by using the Pierce RNA 3^′^ End Desthio-biotinylation Kit (Pierce, Thermo). The Pierce Magnetic RNA-Protein Pull-Down Kit was used to perform the RNA pull-down assays according to the manufacturer’s instructions (Pierce, Thermo).

### Statistical analysis

Statistical analyses were carried out with GraphPad Prism5 (GraphPad Software, La Jolla, USA). The statistical significance of distinctions between various groups was calculated by Student’s *t*-test or a Chi-square test, as suitable. All data were represented as means ± S.D., were counted, and two-sided *P*-values of 0.05 were considered for statistical significance.

## Electronic supplementary material

The list of primers and siRNA sequence

mRNAs increased abundance (≥1.5-fold) in *SNHG1*-knockdown HuCCT1 cells

*SNHG1* and EZH2 do not affect each other in vivo and vitro

Ssupplementary Figure
