# Supplementary material for: Epigenetic silencing of tumor suppressor gene CDKN1A by oncogenic long non-coding RNA SNHG1 in cholangiocarcinoma
Source: Cell Death Dis. 2018 Jul 3;9(7):746. doi: 10.1038/s41419-018-0768-6 (PMC6030364; doi:10.1038/s41419-018-0768-6)
Supplement: Supplementary file 1 — The list of primers and siRNA sequence [file 41419_2018_768_MOESM1_ESM.docx]

| \| **Human qRT-PCR** \| \| \|  \| \| --- \| --- \| --- \| --- \| \| **GENE** \| **Forward primer** \| \| **Reverse primer** \| \| SNHG1 \| ACAGCAGTTGAGGGTTTGCT \| \| GGGCCTGGATCATGTAAGAA \| \| EZH2 \| TGCACATCCTGACTTCTGTG \| \| AAGGGCATTCACCAACTCC \| \| IL32 \| CTCAGTGGAGCTGGGTCATC \| \| CCTGTCCACGTCCTGATTCT \| \| IL11 \| GAGGGCGATTTGTCTGAGAG \| \| GCAACAGAGCGAGACTCCA \| \| LRIG1 \| GCCTGGAAGAAAGACAATGAA \| \| GAGCCAAAGTGGTTGGTGAT \| \| CMTM3 \| TGCTGGCCTTGTACTTCCTC \| \| GCAAACACGATGGTAGCAAA \| \| CDKN1A \| AAAGGCCCGCTCTACATCTT \| \| ATGCCCAGCACTCTTAGGAA \| \| PIK3IP1 \| AGCCCACCCTCCTACAGCTA \| \| GCAATGTTTGGAAGCCCTTA \| \| CD82  G0S2  GDF15  ADAM19 \| CATCCTGGCTAACATGGTGA  GACCGAGAGAGAGGAATGGAGAG  GAGGTGCAAGTGACCATGTG  AAGTACCATGACAACGCCCAAT \| \| GGAGTGCAGTAGCACGATCTC  TTTGGTGGATGCTTGTGGTA  CAGTGGCAGTCTTTGGCTAAC  CATTCTCGGAGTGGTCCATGT \| \| U1 \| GGGAGATACCATGATCACGAAGGT \| \| CCACAAATTATGCAGTCGAGTTTCCC \| \| GAPDH \| AGCCACATCGCTCAGACAC \| \| GCCCAATACGACCAAATCC \| \| **ChIP primers** \|  \| \|  \| \| CDKN1A \| CCTCCTTCTTCAGGCTTGGG \| \| CAGGCAGCATAGGGATGGAG \| \| **Sequences for siRNAs** \| \| \| \| \| SNHG11# \| CCUUAAAGUGUUAGCAGACACAGAU \| \| AUCUGUGUCUGCUAACACUUUAAGG \| \| SNHG1 2# \| CACUUCGUGUCUGUUCCUCUGUAUA \| \| UAUACAGAGGAACAGACACGAAGUG \| \| si-EZH2 \| GAGGUUCAGACGAGCUGAUUU \| \| AUCAGCUCGUCUGAACCUCUU \| \| **ShSNHG1:** **Sequences cloned into pENTR™/U6 vector** \| \| \| \| \| CACCGGCCAGCACCTTCTCTCTAAACGAATTTAGAGAGAAGGTGCTGGCC \| \| AAAAGGCCAGCACCTTCTCTCTAAATTCGTTTAGAGAGAAGGTGCTGGCC \| \| |
| --- | --- | --- | --- | --- | --- | --- | --- | --- | --- | --- | --- | --- | --- | --- | --- | --- | --- | --- | --- | --- | --- | --- | --- | --- | --- | --- | --- | --- | --- | --- | --- | --- | --- | --- | --- | --- | --- | --- | --- | --- | --- | --- | --- | --- | --- | --- | --- | --- | --- | --- | --- | --- | --- | --- | --- | --- | --- | --- | --- | --- | --- | --- | --- | --- | --- | --- | --- | --- | --- | --- | --- | --- | --- | --- | --- | --- | --- | --- | --- | --- | --- | --- | --- | --- |
