# Supplementary material for: Epigenetic silencing of tumor suppressor gene CDKN1A by oncogenic long non-coding RNA SNHG1 in cholangiocarcinoma
Source: Cell Death Dis. 2018 Jul 3;9(7):746. doi: 10.1038/s41419-018-0768-6 (PMC6030364; doi:10.1038/s41419-018-0768-6)
Supplement: Supplementary file 2 — mRNAs increased abundance (≥1.5-fold) in SNHG1-knockdown HuCCT1 cells [file 41419_2018_768_MOESM2_ESM.docx]

| \| AccID \| Log2FC \| FDR \| Style \| KeggID \| Description \| \| --- \| --- \| --- \| --- \| --- \| --- \| \| ANGPTL2 \| 3.271 \| 0.000 \| up \| hsa:23452 \| angiopoietin-like 2 \| \| IL32 \| 6.375 \| 0.000 \| up \| hsa:9235 \| interleukin 32 \| \| IL11 \| 4.956 \| 0.000 \| up \| hsa:3589 \| interleukin 11 \| \| C20orf195 \| 3.703 \| 0.000 \| up \| hsa:79025 \| chromosome 20 open reading frame 195 \| \| GCNT3 \| 3.666 \| 0.000 \| up \| hsa:9245 \| glucosaminyl (N-acetyl) transferase 3, mucin type \| \| C1orf106 \| 3.650 \| 0.000 \| up \| hsa:55765 \| chromosome 1 open reading frame 106 \| \| GBP1 \| 3.379 \| 0.000 \| up \| hsa:2633 \| guanylate binding protein 1, interferon-inducible \| \| LRIG1 \| 2.704 \| 0.000 \| up \| hsa:26018 \| leucine-rich repeats and immunoglobulin-like domains 1 \| \| G0S2 \| 2.165 \| 0.000 \| up \| hsa:50486 \| G0/G1 switch 2 \| \| CMTM3 \| 2.158 \| 0.000 \| up \| hsa:123920 \| CKLF-like MARVEL transmembrane domain containing 3 \| \| GDF15 \| 2.131 \| 0.000 \| up \| hsa:9518 \| growth differentiation factor 15 \| \| CDKN1A \| 2.001 \| 0.000 \| up \| hsa:1026 \| cyclin-dependent kinase inhibitor 1A (p21, Cip1) \| \| PIK3IP1 \| 1.718 \| 0.000 \| up \| hsa:113791 \| phosphoinositide-3-kinase interacting protein 1 \| \| CD82 \| 1.683 \| 0.000 \| up \| hsa:3732 \| CD82 molecule \| \| TSPAN12 \| -1.714 \| 0.000 \| down \| hsa:23554 \| tetraspanin 12 \| \| TOMM34 \| -1.780 \| 0.000 \| down \| hsa:10953 \| translocase of outer mitochondrial membrane 34 \| \| CIZ1 \| -1.801 \| 0.000 \| down \| hsa:25792 \| CDKN1A interacting zinc finger protein 1 \| \| SCAMP1 \| -1.846 \| 0.000 \| down \| hsa:9522 \| secretory carrier membrane protein 1 \| \| HMGN2 \| -1.950 \| 0.000 \| down \| hsa:3151 \| high mobility group nucleosomal binding domain 2 \| \| GAS1 \| -1.968 \| 0.000 \| down \| hsa:2619 \| growth arrest-specific 1 \| \| PEG10 \| -2.024 \| 0.000 \| down \| hsa:23089 \| paternally expressed 10 \| \| TIMP3 \| -2.081 \| 0.000 \| down \| hsa:7078 \| TIMP metallopeptidase inhibitor 3 \| \| MUC16 \| -2.138 \| 0.000 \| down \| hsa:94025 \| mucin 16, cell surface associated \| \| TNFSF10 \| -2.448 \| 0.000 \| down \| hsa:8743 \| tumor necrosis factor (ligand) superfamily, member 10 \| |
| --- | --- | --- | --- | --- | --- | --- | --- | --- | --- | --- | --- | --- | --- | --- | --- | --- | --- | --- | --- | --- | --- | --- | --- | --- | --- | --- | --- | --- | --- | --- | --- | --- | --- | --- | --- | --- | --- | --- | --- | --- | --- | --- | --- | --- | --- | --- | --- | --- | --- | --- | --- | --- | --- | --- | --- | --- | --- | --- | --- | --- | --- | --- | --- | --- | --- | --- | --- | --- | --- | --- | --- | --- | --- | --- | --- | --- | --- | --- | --- | --- | --- | --- | --- | --- | --- | --- | --- | --- | --- | --- | --- | --- | --- | --- | --- | --- | --- | --- | --- | --- | --- | --- | --- | --- | --- | --- | --- | --- | --- | --- | --- | --- | --- | --- | --- | --- | --- | --- | --- | --- | --- | --- | --- | --- | --- | --- | --- | --- | --- | --- | --- | --- | --- | --- | --- | --- | --- | --- | --- | --- | --- | --- | --- | --- | --- | --- | --- | --- | --- | --- |
