# Supplementary figures and images for: Epigenetic silencing of tumor suppressor gene CDKN1A by oncogenic long non-coding RNA SNHG1 in cholangiocarcinoma
Source: Cell Death Dis. 2018 Jul 3;9(7):746. doi: 10.1038/s41419-018-0768-6 (PMC6030364; doi:10.1038/s41419-018-0768-6)

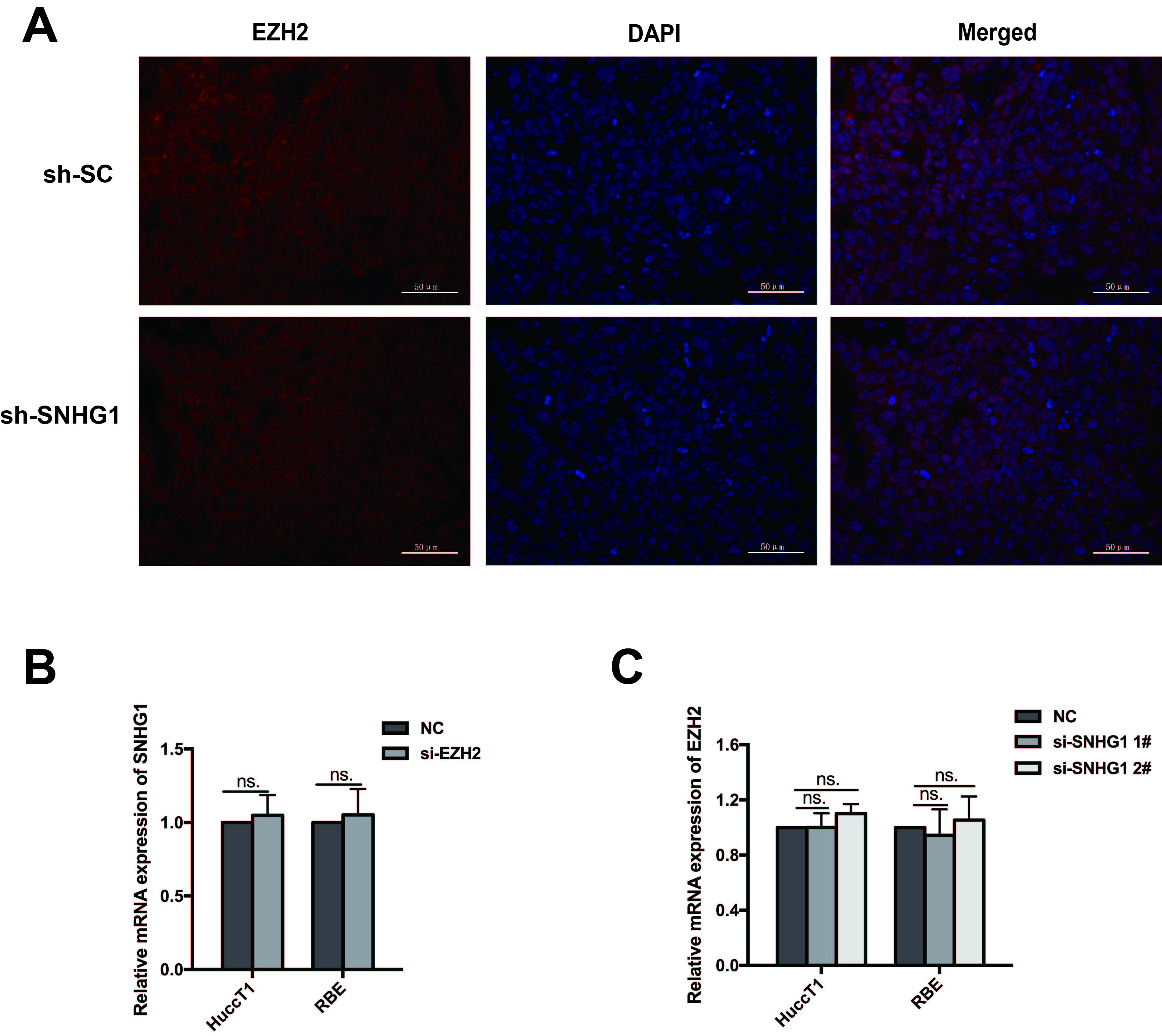

Supplement: Supplementary file 3 — SNHG1 and EZH2 do not affect each other in vivo and vitro [file 41419_2018_768_MOESM3_ESM.jpg]
