# Supplementary material for: Epigenetic silencing of tumor suppressor gene CDKN1A by oncogenic long non-coding RNA SNHG1 in cholangiocarcinoma
Source: Cell Death Dis. 2018 Jul 3;9(7):746. doi: 10.1038/s41419-018-0768-6 (PMC6030364; doi:10.1038/s41419-018-0768-6)
Supplement: Supplementary file 4 — Ssupplementary Figure [file 41419_2018_768_MOESM4_ESM.docx]

**Fig S1. SNHG1 and EZH2 do not affect each other in *vivo* and *vitro*.**

(A) Immunofluorescence was taken to explore if the expression of EZH2 have been changed when knockdown of SNHG1 in vivo. (B) The altered mRNA level of SNHG1 by qRT-PCR when knockdown of EZH2 in CCA cell lines. (C) The altered mRNA level of EZH2 by qRT-PCR when knockdown of SNHG1in CCA cell lines.
